# IFN-γ reprograms cardiac microvascular endothelial cells to mediate doxorubicin transport and influences the sensitivity of mice to doxorubicin-induced cardiotoxicity

**DOI:** 10.1038/s12276-024-01389-7

**Published:** 2025-01-22

**Authors:** Haoyu Ji, Wenya Ma, Xu Liu, Hongyang Chen, Yining Liu, Zhongyu Ren, Daohong Yin, Ao Cai, Zizhen Zhang, Xin Wang, Wei Huang, Leping Shi, Yanan Tian, Yang Yu, Xiuxiu Wang, Yang Li, Yu Liu, Benzhi Cai

**Affiliations:** 1 Department of Pharmacy at The Second Affiliated Hospital, and Department of Pharmacology at College of Pharmacy (The Key Laboratory of Cardiovascular Medicine Research, Ministry of Education), Harbin Medical University, Harbin, P. R. China; 2 Department of Laboratory Medicine at The Fourth Affiliated Hospital, Harbin Medical University, Harbin, P. R. China; 3 Research Unit of Health Sciences and Technology, Faculty of Medicine University of Oulu, Finland; Research Center for Innovative Technology of Pharmaceutical Analysis, College of Pharmacy, Harbin Medical University, Heilongjiang, P. R. China

**Keywords:** Experimental models of disease, Data processing

## Abstract

Doxorubicin (DOX) is a first-line chemotherapy agent known for its cardiac toxicity. DOX-induced cardiotoxicity (DIC) severely limits the use for treating malignant tumors and is associated with a poor prognosis. The sensitivity to DIC varies among patients, but the precise mechanisms remain elusive. Here we constructed a mouse model of DIC using DOX to investigate potential mechanisms contributing to the differential susceptibility to DIC. Through surface-enhanced Raman spectroscopy and single-cell RNA sequencing, we explored the mechanisms underlying DIC phenotypic variations. In vitro and in vivo studies with small-molecule drugs were conducted. DIC-insensitive mice displayed preserved ejection fractions, lower DOX levels in cardiac tissues and higher levels in the serum. Single-cell RNA sequencing revealed differences of gene expression in cardiac endothelial cells between DIC-insensitive and DIC-sensitive groups. The expression of IFN-γ pathway-related genes was high in DIC-insensitive mice. IFN-γ administration decreased the DOX distribution in cardiac tissues, whereas PPAR-γ activation increased DIC susceptibility. IFN-γ stimulation upregulated P-glycoprotein expression, leading to increased DOX efflux and DIC insensitivity. Our model provides insights into the mechanisms of DIC sensitivity and potential preventive strategies.

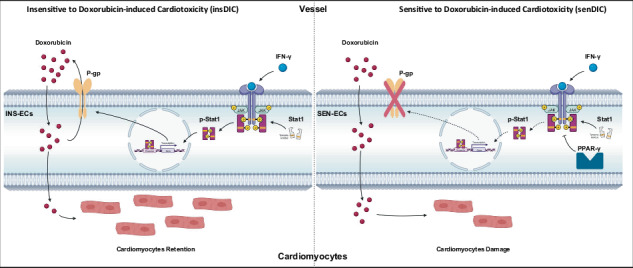

## Mechanisms behind doxorubicin sensitivity revealed in mice

Doxorubicin is a powerful cancer drug, but it can harm the heart, leading to a condition called doxorubicin-induced cardiotoxicity (DIC). Some people are more affected by DIC than others, and scientists want to understand why. They found that the heterogeneity observed among endothelial cells (ECs) plays a potential role in determining DIC sensitivity. In mice less sensitive to DIC, reprogramming of ECs increases levels of P-glycoprotein (P-gp), which helps to pump drugs out of cells. They discovered that activating a pathway involving IFN-γ increased P-gp levels, reducing heart damage. Conversely, activating another pathway, PPAR-γ, decreased P-gp levels and increased heart damage. These findings provide new insights into DIC pathogenesis and suggest that boosting P-gp in ECs could be a new strategy to protect against DIC. This summary was initially drafted using artificial intelligence, then revised and fact-checked by the author.

## Introduction

Doxorubicin (DOX), a potent broad-spectrum anthracycline antibiotic, is widely used in cancer treatment. However, its clinical application is restricted by serious cardiotoxicity, which can lead to left ventricular dysfunction and eventually heart failure, a condition known as DOX-induced cardiotoxicity (DIC)^1^. The development of DIC is closely associated with a poor prognosis and mortality in cancer survivors. Notably, individual differences in the occurrence of DIC have been observed among patients treated with DOX, where some patients exhibit impaired cardiac function, while others are not sensitive^2^. These individual differences are probably influenced by a complex interplay of genetic and environmental factors. Similar variations have been noted among individuals of the same inbred strain of mice^3^. Traditionally, individual differences have often been considered as sources of variability or potential measurement challenges in animal experiments^4^. However, an increasing number of studies are exploring the use of model animals to better understand the reason for the differences as they relate to human health^5,6^. In recent years, numerous studies have reported potential molecular mechanisms underlying DIC, including oxidative stress, impaired calcium homeostasis, transcriptional dysregulation mediated by Top2β inhibition, and mitochondrial damage^7^. However, the reasons for the differences in susceptibility to DIC have not yet been elucidated completely. Defining the potential mechanism of individual differences in DIC is important for its prevention.

Cardiac endothelial cells (ECs), the most abundant noncardiomyocyte type in the heart, play vital roles in various physiological and pathological processes, including angiogenesis, myocardial fibrosis, hypertrophy, and cardiomyocyte (CM) apoptosis^8^. ECs form tight connections that create a physical barrier between circulating blood and CMs, safeguarding CMs from harmful substances and releasing paracrine factors to maintain CM function^9^. Wilkinson et al. demonstrated that the expression of the tight junction protein ZO-1 was reduced in DOX-treated human ECs, resulting in increased microvascular permeability and prolonged exposure of CMs to DOX^10^. In addition, DOX inhibits the secretion of endothelial-derived paracrine molecules such as ET-1, NO, PGI2 and NRG-1, which act on CMs, influencing myocardial function, survival and adaptation to environmental stressors^11–13^. Although the close relationship between CMs and ECs is well established, our understanding of the molecular mechanisms through which DOX affects ECs in DIC remains limited. Whether DOX-mediated alterations in EC function are responsible for differences in DIC sensitivity needs to be further identified.

P-glycoprotein (P-gp) is an ATP-binding cassette superfamily transporter present in the blood‒brain barrier (BBB), where it restricts the entry of exogenous drugs into the brain^14^. P-gp is able to recognize a broad range of substrates, and alterations in its function can considerably impact the concentration, toxicity and efficacy of drugs^15^. For example, increased P-gp function reduces drug concentrations in the brain, leading to drug resistance in individuals with diseases such as epilepsy and acquired immune deficiency syndrome^16^. Conversely, decreased P-gp function can increase drug or toxin levels in the brain, potentially inducing neurotoxicity^17^. DOX has been identified as a substrate for P-gp. The exposure of cancer cells to DOX stimulates P-gp expression, which consequently mediates chemotherapy resistance^18,19^. However, whether DOX-induced change in P-gp expression influence DOX distribution in heart by mediating EC function and whether differences in P-gp expression contribute to DIC sensitivity remain unexplored.

In this study, we aimed to investigate the regulatory roles of P-gp and ECs in a DIC mouse model and elucidate its underlying mechanisms. Our results revealed that the expression of P-gp in microvascular ECs was increased in the DIC-insensitive group. Mechanistically, the DIC-insensitive group exhibited notale activation of interferon-γ (IFN-γ), which upregulated P-gp expression and enhanced DOX efflux by activating the STAT1 pathway, ultimately leading to DIC insensitivity in mice. Conversely, peroxisome proliferator-activated receptor-γ (PPAR-γ) signaling was predominantly activated in DIC-sensitive mice, which downregulated P-gp expression by blocking the IFN-γ pathway and ultimately aggravated the myocardial toxicity of DOX.

## Materials and Methods

All the data needed to evaluate the conclusions here are presented in the Article or its Supplementary Information. Additional data related to this Article may be requested from the authors. Detailed descriptions of the materials and methods can also be found in the Supplementary Information.

### Animals

Adult male C57BL/6J mice (8–10 weeks old, weighing 20–22 g) were provided by Changsheng Biotechnology. Mice were fed in a facility with a 12 h light/12 h dark cycle at 23 ± 3 °C and 30–70% humidity. Food and water were freely accessible to the mice. Male mice were used to avoid the influence of estrogen fluctuations in female mice and to ensure the consistency of the results of our study. Echocardiography measurements were performed by a single experienced operator in a blinded manner. After the baseline echocardiographic measurements, the ejection fraction (EF) and fractional shortening were measured. Animals with an EF ≤60% or fractional shortening ≤40% were eliminated. The remaining mice were randomly assigned to the different experimental groups. The operators responsible for the experimental procedures were blinded to the group allocation, and the operators responsible for data analysis were blinded and unaware of group allocation throughout the experiments.

### In vivo drug treatment

A cumulative dose of 12.3 mg/kg DOX (cat. no. MB1087, Dalian Meliun Biotechnology, dissolved in sterile saline) was administered via two intraperitoneal (i.p.) injections (6.15 mg/kg on days 0 and 3) to mimic human therapeutic regimens, and the control group was injected with an equal amount of normal saline. The survival of the mice was determined every day, and echocardiography was performed to assess cardiac function and used for further experiments. We selected the recombinant IFN-γ protein (cat. no. ab259378, Abcam) and fludarabine (Flud; cat. no. HY-B0069, MCE) as an agonist and inhibitor of IFN-γ pathway, respectively, to investigate the role of the IFN-γ pathway in DIC. The mice were injected 30 min before the DOX injection (recombinant IFN-γ protein 100 μg/kg in 1% bovine serum albumin; Flud 0.8 mg/kg in the in vivo administration solvent). Solutions of the in vivo administration solvent (10% dimethyl sulfoxide, 40% PEG300, 5% Tween-80 and 45% saline) were mixed with agents to dissolve them completely. Rosiglitazone (Rosi; cat. no. R2408, MCE) and T0070907 (T007; cat. no. HY-13202, MCE) were selected as an agonist and inhibitor of the PPAR-γ pathway, respectively, to investigate the role of the PPAR-γ pathway in DIC. The mice were injected 30 min before the DOX injection (recombinant Rosi 5 mg/kg in the in vivo administration solvent, daily gavage; T007 1 mg/kg in the in vivo administration solvent). Tariquidar (Tar; cat. no. XR9576, MCE), an inhibitor of P-gp, was used to study the role of P-gp in DIC. The mice were injected 30 min before the DOX injection (8 mg/kg in the in vivo administration solvent). In particular, all the mice in the Tar combined with DIC model group died on the seventh day, and thus we chose the fifth day as the time point for studying this model.

### Statistical analysis

The data were statistically analyzed using GraphPad Prism 9.5 software, and the data are presented as the mean ± s.e.m. An unpaired (two-tailed) Student’s test was used when analyzing two datasets. For the analysis of data from multiple groups, one-way analysis of variance was used (*P < 0.05, **P < 0.01, ***P < 0.001).

## Results

### Different sensitivities of mice to DIC

**Fig. 1:**
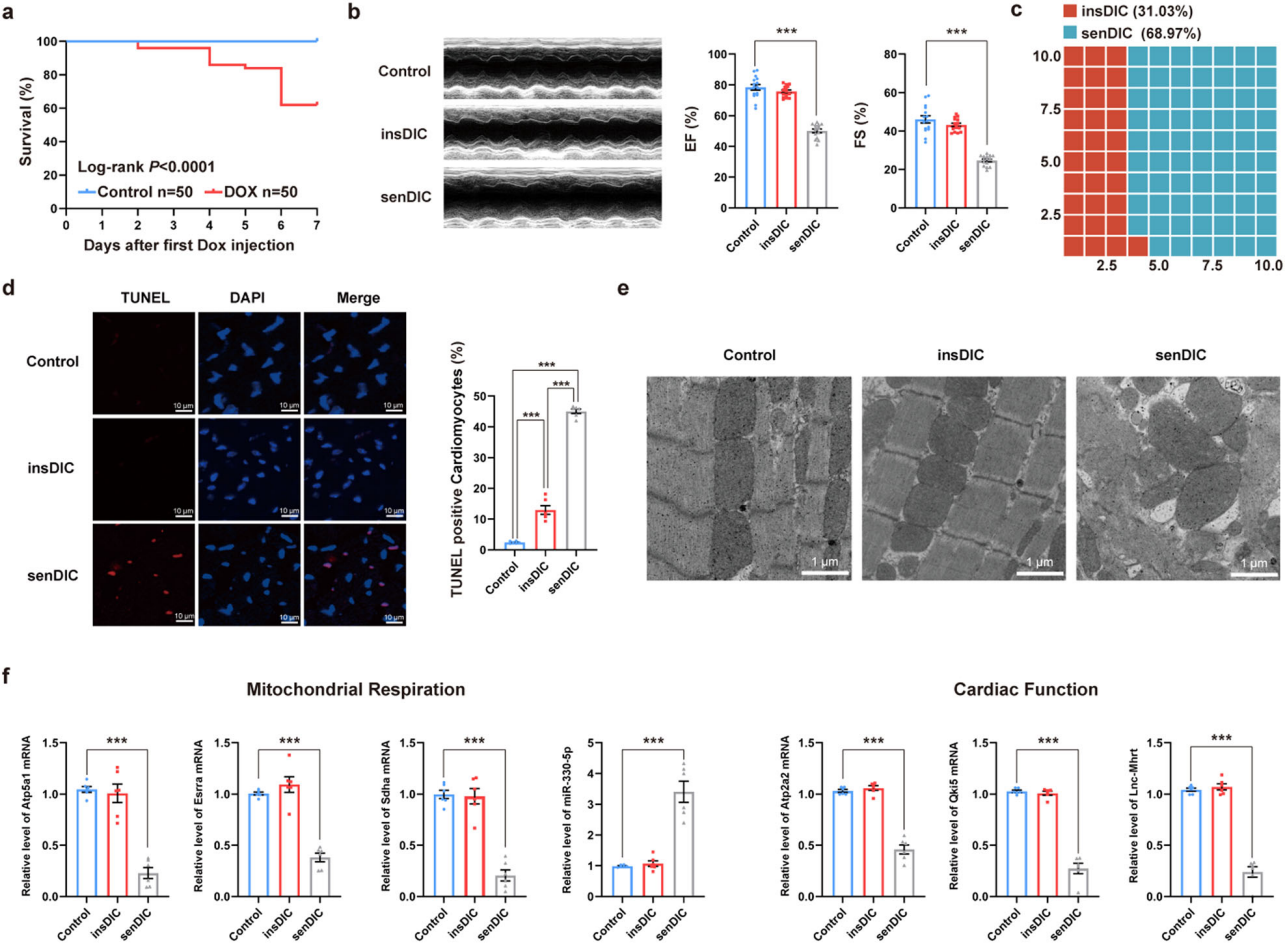
Different sensitivities to DIC are observed in mice. **a**, Kaplan–Meier survival curves of mice pretreated with saline (control) or 12.3 mg/ml (clinical dose) DOX (i.p.) on days 0 and 3 (*n* = 50 mice per group). **b**, Representative echocardiographic photographs showing the cardiac function of the control, insDIC and senDIC mice. ***P < 0.001. *n* = 15. **c**, The proportions of mice in the insDIC group and senDIC group were analyzed. **d**, Representative images of TUNEL-stained sections of heart tissues from control mice and insDIC or senDIC mice. ***P < 0.001. Scale bars, 10 µm. *n* = 6. **e**, Representative TEM images of heart tissues from control and insDIC or senDIC mice. Scale bars, 1 µm. *n* = 3. **f**, The expression of DIC markers (*Atp5a1*, *Esrra*, *Sdha*, miR-330-5p, *Atp2a2*, *Qki5* and lnc-Mhrt) in control mice and insDIC or senDIC mice was detected via qRT‒PCR. ***P < 0.001; n.s., not significant. *n* = 6. All the data are presented as the mean ± s.e.m.

Clinical evidence has indicated that patients treated with DOX exhibit different sensitivities to DIC^20^. Similar observations have been made in animal studies involving DOX-treated hearts. However, the deep explanation for this phenomenon remains limited. Here, we aimed to explore the fundamental mechanism of non-sensitivity to DIC, providing pharmacological interventions for addressing cardiotoxicity in clinical settings (Supplementary Scheme 1). The incidence of DIC in patients receiving therapeutic doses of DOX has been reported to range from 10% to 48% in early studies^21^. We constructed a mouse model of DIC by intraperitoneally injecting a clinically relavent dose (12.3 mg/kg) of DOX to investigate the differences in DIC sensitivity (Fig. 1a)^22^. Echocardiography measurements conducted on day 7 of DOX treatment revealed that approximately 31.03% of the mice presented a preserved left ventricular ejection fractions (EF ≥60%) and were classified as insensitive to DIC (insDIC). By contrast, about 68.96% of the mice displayed a significant decrease in the EF (EF <60% and a reduction of more than 10% in EF)^23^, which were labeled as sensitive to DIC (senDIC) (Fig. 1b, c). We subsequently evaluated cardiac injury by assessing CM apoptosis, mitochondrial and CM morphology, and indicators associated with DOX cardiotoxicity across different groups. Terminal deoxynucleotidyl transferase-mediated dUTP nick-end labeling (TUNEL) staining revealed a substantially greater number of apoptotic CMs in the senDIC group than in the control and insDIC groups (Fig. 1d). Transmission electron microscopy (TEM) revealed severe myocardial injury in the senDIC group, as indicated by damaged myofilaments and irregularly shaped mitochondria. The insDIC group presented slightly relaxed muscle filaments and circular or elliptical mitochondria, indicating mild cardiac damage (Fig. 1e). Furthermore, we analyzed the expression of key indicators of DIC, including *Atp5a1*, *Esrra*, *Sdha*, *Atp2a2*, *Qki5*, lnc-Mhrt, and miR-330-5p^24–27^. The levels of these markers were significantly altered in the senDIC group, which is consistent with previous reports, whereas no marked alterations were observed in the insDIC group (Fig. 1f). These results indicate that the senDIC mice exhibited obvious DIC, whereas the insDIC mice demonstrated resistance to DIC.

### Reduced myocardial distribution of DOX in insDIC mice

**Fig. 2:**
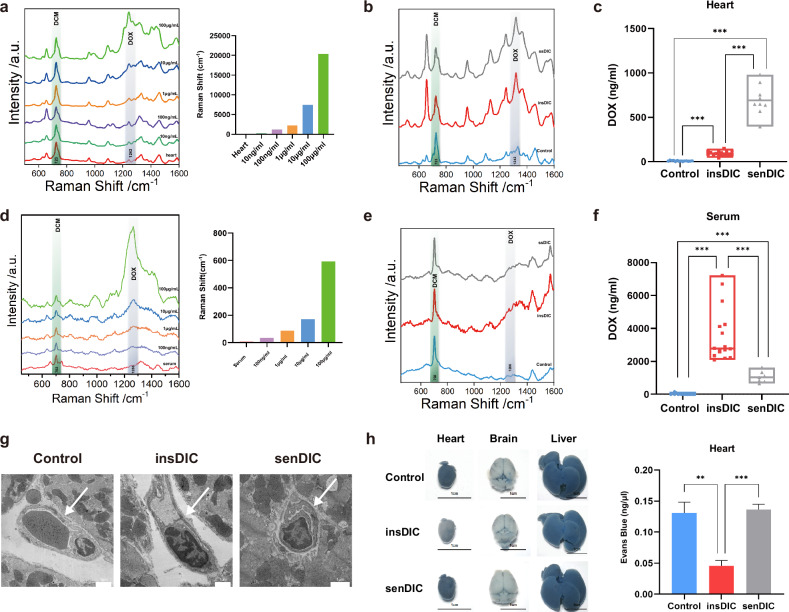
A lower myocardial distribution of DOX is associated with the insensitivity of DIC. **a**, Left: SERS spectra of heart tissues from mice treated with different concentrations of DOX. Right: changes in the heart DOX *I*_1242_/*I*_723_ ratio with the corresponding concentration in mice.**b**, SERS spectra of the DOX concentration in heart tissues from mice in the control (blue line), insDIC (red line) and senDIC (gray line) groups. Experimental conditions: laser power of 0.95 mW (632.8 nm); 5 scans of 10 s. **c**, Quantitative analysis of the DOX concentration in heart tissues from the mice in each group. ***P < 0.001. **d**, Left: SERS spectra of serum samples from mice treated with different concentrations of DOX. Right: changes in the serum DOX *I*_1266_/*I*_702_ ratio with the corresponding concentration in mice. **e**, SERS spectra of serum DOX concentrations in the control (blue line), insDIC (red line) and senDIC (gray line) groups. The experimental conditions included a laser power of 0.95 mW (632.8 nm) and 5 scans of 10 s. **f**, Quantitative analysis of the serum DOX concentration in each group. ***P < 0.001. **g**, Representative TEM images of cardiac vessels from control and insDIC or senDIC mice. Scale bars, 1 µm. *n* = 3. **h**, Overall morphology and concentrations of the heart, liver and brain in the control, insDIC and senDIC groups after Evans blue staining. **P < 0.01, ***P < 0.001. *n* = 6. All the data are presented as the mean ± s.e.m.

Since the cardiotoxicity induced by DOX on CMs is dose dependent^28^, we further investigated whether the different sensitivities to DIC could be attributed to variations in the DOX content within heart tissues. As a method to validate this hypothesis, we used surface-enhanced Raman spectroscopy (SERS) to detect the distribution of DOX in both mouse heart tissues and serum. We first detected the Raman signals of DOX at various concentrations in cardiac tissues and observed a direct correlation between the characteristic peak intensity of DOX at 1,242 cm^−1^ and the amount of DOX present (Fig. 2a)^29^. We subsequently measured the DOX concentration in the myocardial tissues of mice with differences in DIC sensitivity and found that the DOX level in the senDIC group was significantly higher than that in the insDIC group (Fig. 2b, c). Conversely, the serum DOX concentration in the insDIC group was obviously higher than that in the senDIC group (Fig. 2d–f). To study if the variations in drug distribution were related to DOX-induced damage to ECs, TEM was used to image three groups of microvascular ECs. The analysis revealed no significant differences in the morphology and integrity of ECs among the groups (Fig. 2g). Furthermore, we conducted Evans blue staining to confirm the differential drug transport capacity in myocardial tissues. The results revealed the differences in the staining of the hearts among the groups, with the insDIC group showing the lighter staining (Fig. 2h). These data indicate that the drug transport ability is different between insDIC and senDIC mice. The serum DOX concentration is high, whereas the heart tissue DOX content is low in insDIC mice, which may contribute to their resistance to DIC. The reason for this shift in transport capacity may be the presence of the blood‒myocardium barrier, but further investigations are needed to elucidate the underlying mechanisms involved.

### Cardiac ECs are reprogrammed in insDIC mice

**Fig. 3:**
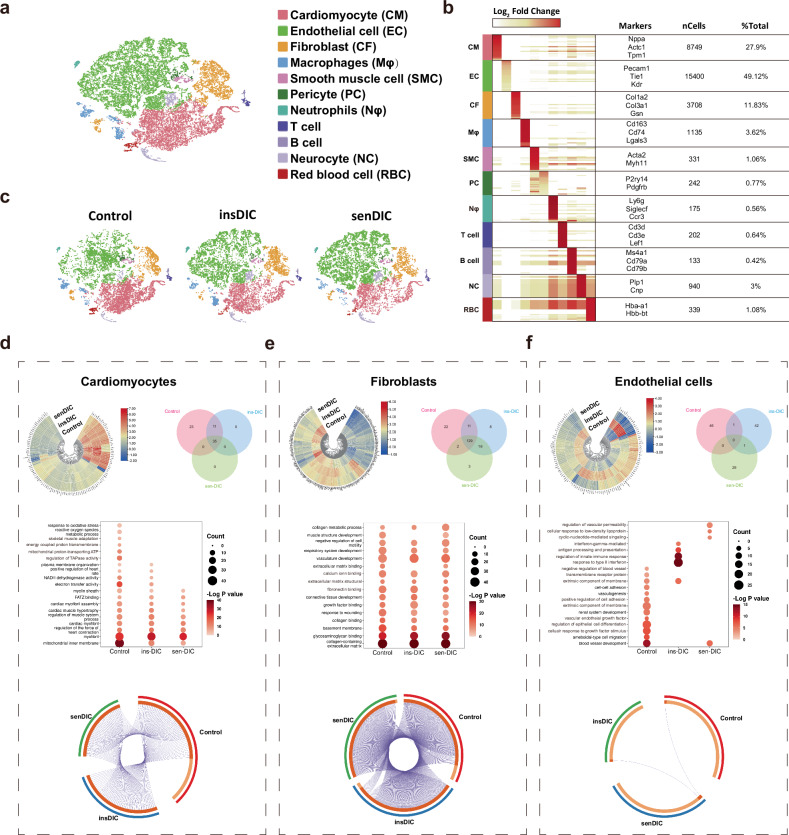
Cardiac ECs are reprogrammed in DIC-insensitive mice. **a**, A t-SNE plot showing the single-cell transcriptomes analyzed in the study, which are color-coded for clusters of different types of cells. **b**, A heatmap showing the mRNAs that were enriched in the top 20 genes of each cell cluster. **c**, A t-SNE plot showing clusters of different types of cellular heterogeneity in different groups. **d**, The ring heatmap shows the differences in the transcriptional profiles of CMs among the three groups. The Venn diagram shows the DEGs in the CMs of the three groups of mice. The correlation ring plot shows the correlation of the transcriptomes among the three groups of mouse CMs. A GO analysis was performed according to the principles of a false discovery rate (FDR) <0.01 and |log_2_fold change| >1 to filter out DEGs from the transcriptional profiles of the three groups of CMs. **e**, The ring heatmap shows the differences in the transcriptional profiles of fibroblasts among the three groups. The Venn diagram shows the DEGs in the fibroblasts from the three groups of mice. The correlation ring plot shows the correlation of the transcriptomes among the three groups of mouse fibroblasts. A GO analysis was performed according to the principles of an FDR <0.01 and |log_2_fold change| >1 to filter out DEGs from the transcriptional profiles of the three groups of fibroblasts. **f**, The ring heatmap shows the differences in the transcriptional profiles of ECs among the three groups. The Venn diagram shows the DEGs in the ECs from the three groups of mice. The correlation ring plot shows the correlation of the transcriptomes among the three groups of mouse ECs. A GO analysis was performed according to the principles of an FDR <0.01 and |log_2_fold change| >1 to filter out DEGs from the transcriptional profiles of the three groups of ECs.

We generated an unbiased single-cell RNA sequencing (scRNA-seq) atlas of mouse hearts from the three groups to elucidate the potential molecular mechanism underlying the difference in DIC in mice. We analyzed 10,709 cells from the control group, 10,278 cells from the insDIC group and 10,367 cells from the senDIC group and identified 17 distinct cell types, including CMs, ECs, fibroblasts, macrophages, smooth muscle cells, T cells and B cells (Fig. 3a). A heatmap was generated to illustrate the marker genes and the top 20 genes with the highest expression levels in each cell type (Fig. 3b). We then visualized the cell populations in the hearts of different groups of mice using *t*-distributed stochastic neighbor embedding (t-SNE). All 17 cell types described earlier were present in the hearts of each mouse group, with some cells exhibiting heterogeneity across the three groups (Fig. 3c). CMs, fibroblasts and ECs constituted the major cellular components of the heart. Further analysis focused on these three cell types to elucidate the molecular mechanism underlying the difference in the DOX distribution.

We conducted circular heatmap analysis and identified differentially expressed genes (DEGs) (*P* < 0.05, |fold change| >1.5) in the control, insDIC and senDIC groups to explore the transcriptional profile of CMs. We identified 69, 46 and 35 DEGs in the control, insDIC and senDIC groups, respectively. Notably, the DEGs in the senDIC group were a subset of those present in the insDIC group (Fig. 3d). We subsequently performed functional correlation loop and Gene Ontology (GO) analyses of these DEGs. The results revealed greater enrichment of specific items related to CMs in the control group. By contrast, the insDIC group presented a relatively lower number of enriched entries, whereas the senDIC group presented the fewest enriched entries among the three mouse groups (Fig. 3d). The GO analysis further revealed enrichment in terms such as myofibrils, inner mitochondrial membranes and electrical conduction activity for all three groups. However, CMs from mice treated with DOX were not enriched in categories such as mitochondrial proton transport, the ATP synthase complex, energy-coupled proton transmembrane transport and the oxidative stress response. In addition, CMs from the senDIC group, which experienced severe heart damage, did not show enrichment in the terms NADH dehydrogenase activity or positive regulation of heart rate (Fig. 3d). The Kyoto Encyclopedia of Genes and Genomes (KEGG) analysis revealed enrichment in pathways associated with dilated cardiomyopathy, myocardial contraction and oxidative phosphorylation in all three groups of CMs. However, certain complexes were not enriched in CMs after DOX administration. The senDIC group displayed no additional enriched pathways, such as thermogenic effects and right ventricular cardiomyopathy (Supplementary Fig. 1a, b). These results indicate that differential gene expression in cardiomyocytes is associated with CM damage, and the degree of injury varies between the senDIC and insDIC groups.

The transcriptional spectrum of cardiac fibroblasts, crucial components of the heart, was analyzed. A circular heatmap was used to identify DEGs (*P* < 0.05, |fold change| >1.5) in each group (Fig. 3e). In the control, insDIC and senDIC groups, 164, 164 and 150 DEGs, respectively, were identified. Among these, 67.54% (129) were common across all groups. Subsequently, functional correlation loop, GO and KEGG analyses revealed significant correlations of terms related to fibroblast function in all groups. The enriched signaling pathways were associated with the typical characteristics and functions of fibroblasts (Fig. 3e); therefore, we did not explore this further.

ECs are another crucial cell type in the heart. The transcriptional spectrum of ECs was analyzed, and DEGs (*P* < 0.05, |fold change| >1.5) were identified in the control, insDIC and senDIC groups (Fig. 3f). These groups exhibited 47, 44 and 30 DEGs, respectively. Interestingly, minimal overlap of DEGs was observed among the three groups, which is highly unusual for the same cell type, arousing our curiosity. A functional correlation analysis of the DEGs in ECs revealed almost no functional correlations among the three groups. The GO analysis revealed that the DEGs in the control group were associated mainly with classic EC characteristics, whereas the DEGs in the insDIC and senDIC groups were associated with immune reprogramming and permeability reprogramming, respectively. The KEGG analysis revealed that the transcriptional profiles and functions of different genes in the ECs differed extensively among the three groups (Supplementary Fig. 1a, b). These findings suggest that ECs in the heart undergo reprogramming after treatment with DOX,and this reprogramming in ECs could be critical for the DOX distribution and DIC sensitivity.

Overall, DOX administration caused CM loss and damage, with senDIC mice experiencing more severe injury than insDIC mice. Pronounced heterogeneity was observed among ECs in the three groups, suggesting their potential role in determining DIC sensitivity.

### DIC sensitivity is associated with different patterns of EC reprogramming

**Fig. 4:**
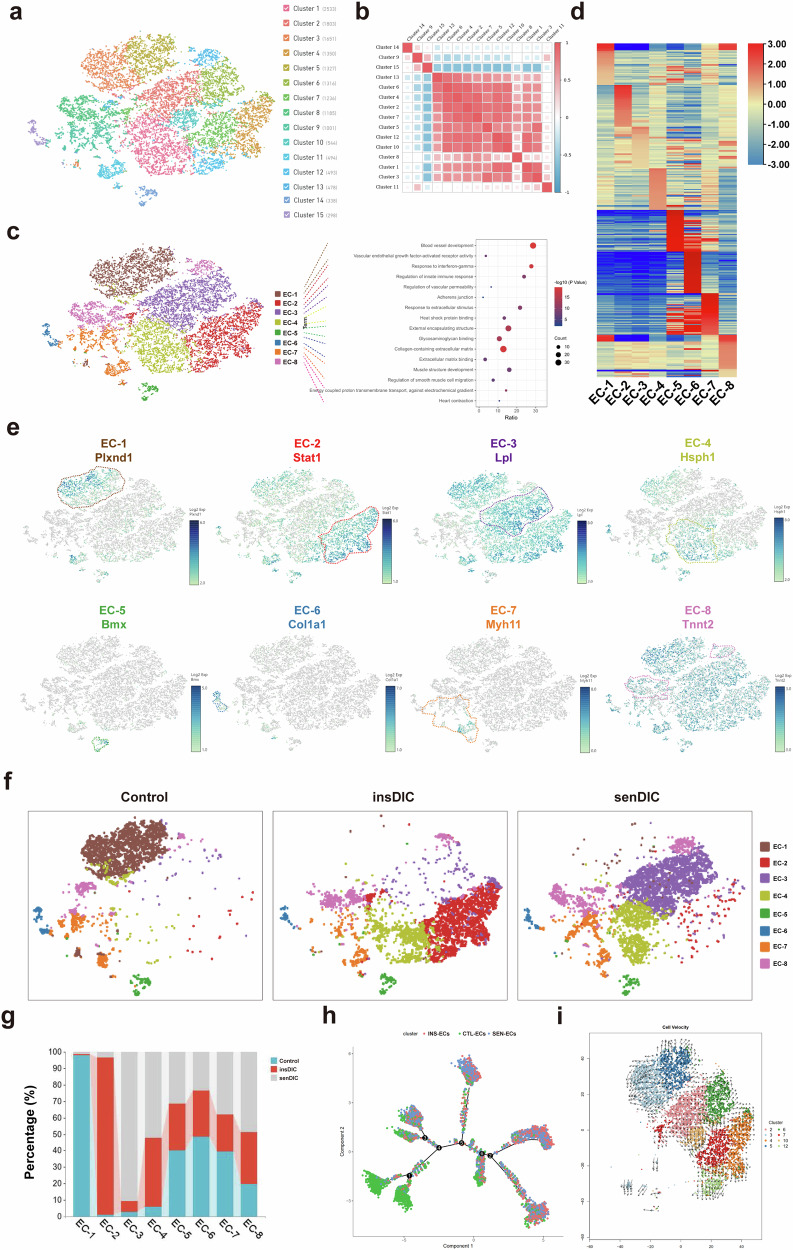
Cardiac microvascular ECs play a key role in determining the sensitivity to DIC. **a**, A t-SNE plot showing the different EC subpopulations analyzed in the study, which are color-coded for clusters of different types of cells. **b**, A correlation heatmap showing correlations among EC subsets. **c**, DEGs were screened from the transcriptional profiles of EC subsets subjected to reclustering and analyzed according to the FDR <0.01 |log_2_fold change| >1 principle of the GO analysis (right), and the EC subsets were named according to the GO results. The results are displayed in a t-SNE diagram (left), and different named EC subsets are distinguished by color. **d**, A heatmap showing mRNAs enriched in the top 50 genes of each EC subset. **e**, t-SNE plots showing marker gene expression in each EC subset. **f**, A t-SNE plot showing clusters of EC subsets with heterogeneity in different groups. **g**, The dynamic changes in the percentage of each class of cells in the total cells from different groups of mice. **h**, Monocle analyses showing CTL-ECs, INS-ECs and SEN-ECs in pseudotime. Each color indicates an EC subset. **i**, RNA velocity analysis was used to estimate single-cell trajectory changes via an analysis of unspliced mRNAs in three subsets of CTL-ECs, INS-ECs and SEN-ECs. Each color indicates a cell cluster.

We further analyzed ECs (Kdr and Pecam1) to investigate the potential impact of EC reprogramming on the DOX distribution^30^. Using t-SNE, we identified 15 subgroups within the EC population (Fig. 4a), which were reclustered to yield 8 distinct EC subpopulations based on transcriptional similarity (Fig. 4b). We performed a GO analysis to delve deeper into the function of each EC subpopulation. Our findings revealed that EC-1 cells were significantly enriched in terms associated with vascular development and vascular endothelial growth factor-activated receptor activity, which aligns with the classical functions of microvascular ECs^30^. The genes associated with EC-2 were enriched in terms related to the IFN-γ regulatory response and the innate immune response, which are closely related to the immune response. EC-3 cells demonstrated a predominance of genes associated with vascular permeability regulation and tight junctions, which are important for drug transport and permeability. Moreover, genes in EC-4 were enriched primarily in terms related to cellular stress, such as the response to extracellular stimuli and heat shock factor binding protein, indicating a stressed state. The gene signature of EC-5 revealed notable enrichment in terms associated with the external encapsulating structure and glycosaminoglycan-binding protein. Specifically, the overexpression of Bmx and Npr3 indicated the identity of these cells as endocardial ECs^31^. EC-6 displayed gene enrichment related to the collagen-containing extracellular matrix and extracellular matrix binding. Notably, it expressed characteristic fibroblast genes, such as *Col1a1* and *Dcn*, suggesting a fibroblast-like EC identity. EC-7 was enriched in genes associated with muscle structural development and the regulation of smooth muscle cell migration, particularly genes such as *Myh11* and *Acta2*, indicating smooth muscle-like EC identity. Finally, EC-8 was enriched in genes related to heart contraction and energy-coupled proton transmembrane transport. It specifically expressed genes such as *Tnnt2* and *Myl2*, which are classic markers of CMs. Therefore, EC-8 could be classified as myocardial-like ECs (Fig. 4c). A heatmap analysis of the top 50 expressed genes in each EC subpopulation highlighted substantial transcriptional disparities among different subpopulations (Fig. 4d). Furthermore, we examined the single-cell expression patterns of marker genes in different subpopulations, which confirmed their high enrichment in the corresponding EC subpopulations and alignment with their specific enriched functions (Fig. 4e). We verified the origin of EC reprogramming, and an analysis of EC subpopulations using t-SNE revealed marked heterogeneity and group preferences (Fig. 4f). A subgroup composition analysis of the hearts of the mice in the different groups revealed that EC-4 to EC-8 did not exhibit significant group preferences. Remarkably, EC-1 was present mainly in the control group, EC-2 was present mainly in the insDIC group, and EC-3 was present mainly in the senDIC group (Fig. 4g). This interesting phenomenon of the group preferences of the EC-1, EC-2 and EC-3 subsets suggests that these three cell subsets are the key cell populations for EC reprogramming after DOX administration. Consequently, we named these subsets the control group-associated ECs (CTL-ECs), insDIC group-associated ECs (INS-ECs) and senDIC group-associated ECs (SEN-ECs), respectively. CTL-ECs are classic microvascular ECs, INS-ECs prevent DOX penetration and SEN-ECs promote DOX penetration. We conducted a pseudotime analysis and RNA velocity analysis to explore the relationships among CTL-ECs, INS-ECs and SEN-ECs. The results demonstrated that both INS-ECs and SEN-ECs differentiated from CTL-ECs independently without any upstream–downstream relationships (Fig. 4h, i). INS-ECs and SEN-ECs play crucial roles in DOX transport and distribution. We analyzed the genes expressed in INS-ECs to further investigate the molecular mechanism underlying INS-EC-induced reprogramming and its impacts on DOX transport and DIC sensitivity. The GO analysis revealed enrichment in functional categories such as the response to IFN-γ, cellular response to IFN-γ, regulation of the innate immune response, and response to viruses. The KEGG analysis highlighted pathways associated with herpes simplex virus type 1 infection, human immunodeficiency virus type 1 infection, influenza A infection and hepatitis C infection (Supplementary Fig. 2a). Therefore, INS-ECs represent a distinct subtype of microvascular ECs characterized by the activation of the IFN-γ pathway. We quantified the levels of IFN-γ in the hearts of the mice from each group, confirming higher expression in the insDIC group, which was consistent with our bioinformatics analysis (Supplementary Fig. 3). In addition, the GO analysis of genes expressed in SEN-ECs revealed enrichment in functional categories related to the regulation of vascular permeability, cell junction assembly, the regulation of metal ion transport, and adherens junctions. The KEGG analysis revealed pathways associated with the PPAR signaling pathway, lipid and atherosclerosis, the adipocytokine signaling pathway and aldosterone synthesis and secretion (Supplementary Fig. 2b). These findings identify SEN-ECs as a distinct subtype of microvascular ECs characterized by the activation of PPAR-γ.

Overall, the results suggest that INS-ECs play a critical role in the DOX distribution in serum and resistance to DIC, whereas SEN-ECs contribute to DOX enrichment in cardiac tissues and DIC development. These findings further indicate that the differences in DOX distribution and cardiac function after DOX administration are attributed to difference in drug transport capacity. INS-ECs may be crucial for DIC resistance, whereas SEN-ECs may contribute to DIC. These two cell subtypes represent distinct microvascular ECs derived from CTL-ECs.

### The DIC incidence increases after blocking the IFN-γ pathway or activating the PPAR-γ pathway

**Fig. 5:**
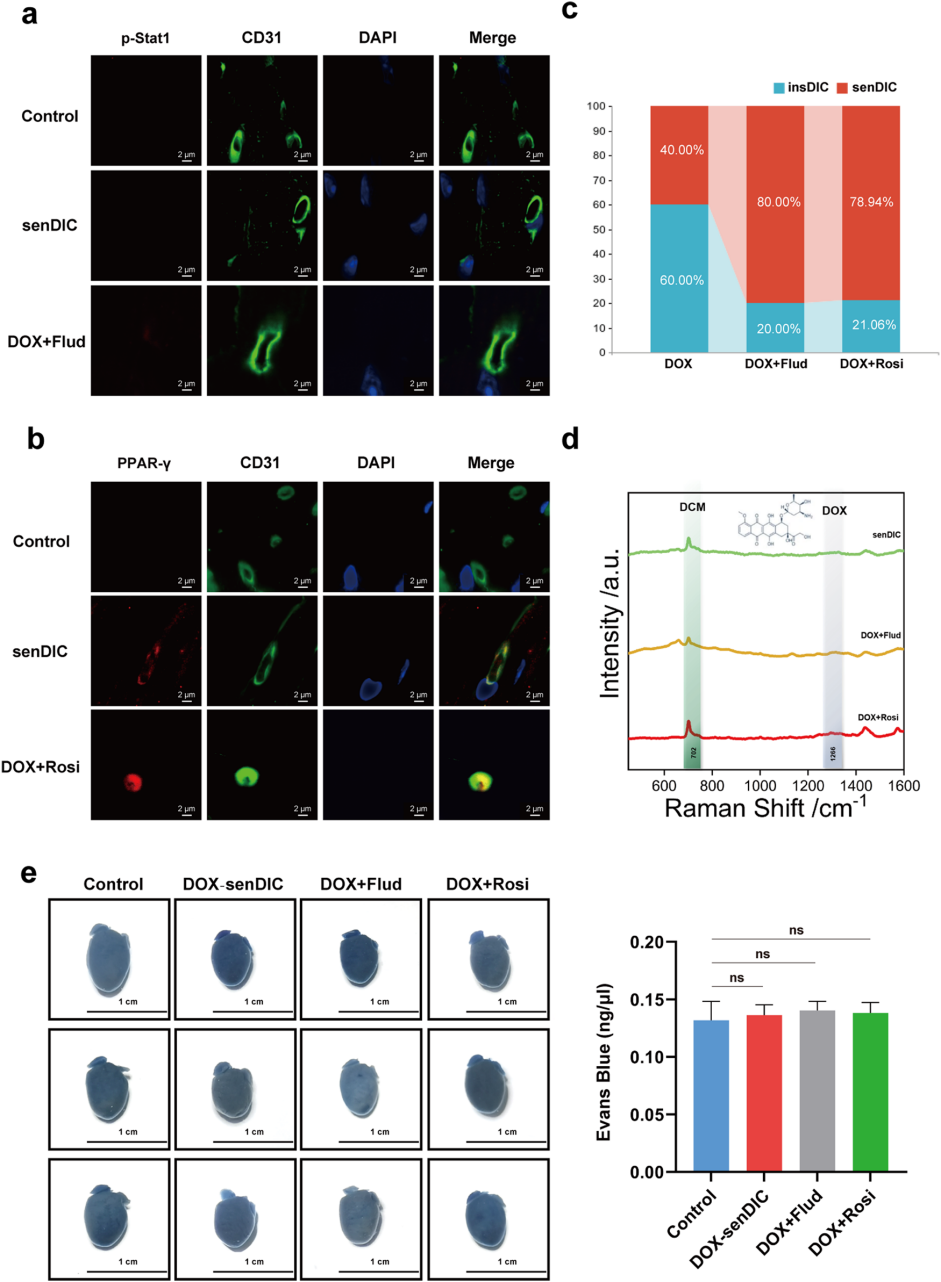
The incidence of DIC is increased by blocking the IFN-γ pathway or activating the PPAR-γ pathway. **a**, Immunofluorescence staining was performed to detect the levels of p-Stat1 in CD31-labeled ECs. Scale bars, 5 µm. **b**, Immunofluorescence staining was performed to detect the expression of PPAR-γ in CD31-labeled ECs. Scale bars, 5 µm. **c**, The proportions of mice in the insDIC group and senDIC group in the DOX group, DOX+Flud group and DOX+Rosi group were analyzed. **d**, SERS spectra of the DOX drug concentration in heart tissues from mice in the senDIC (green line), DOX+Flud (yellow line) and DOX+Rosi (red line) groups. Experimental conditions: laser power of 0.95 mW (632.8 nm); 5 scans of 10 s. **e**, The overall morphology of the hearts of the control, senDIC, DOX+Flud and DOX+Rosi groups after Evans blue staining. n.s., not significant. Scale bars, 1 cm. *n* = 6. All the data are presented as the mean ± s.e.m.

We conducted staining for p-Stat1 and PPAR-γ in CD31-positive cardiac microvascular ECs from the senDIC group of mice to examine the involvement of the IFN-γ and PPAR-γ pathways in DIC sensitivity. We observed PPAR-γ activation but not p-Stat1 activation in these cells, suggesting that the PPAR-γ pathway may contribute to DOX accumulation in cardiac tissue and the development of DIC (Fig. 5a, b). We administered the PPAR-γ agonist Rosi and the p-Stat1 inhibitor Flud to DOX-treated mice to validate these findings. The administration of these drugs to DOX-treated mice significantly increased PPAR-γ expression and inhibited p-Stat1 expression in cardiac ECs (Fig. 5a, b). Doppler echocardiography revealed a higher incidence of DIC in mice treated with Rosi (78.94% in the senDIC group) or Flud (80% in the senDIC group) (Fig. 5c). We measured the DOX content in serum and cardiac tissue using SERS to assess the impacts of DIC sensitizers on the DOX distribution. The sensitizers caused an increase in the DOX concentration in the cardiac tissues and decreased the DOX concentration in the serum (Fig. 5d). In addition, we performed Evans blue staining to investigate the ability of cardiac tissue to take up exogenous substances in the presence of DIC sensitizers and observed increased uptake of the dye by the tissue (Fig. 5e). Therefore, our results indicate that activation of the PPAR-γ pathway and inhibition of the IFN-γ pathway may contribute to DIC development and increased DOX accumulation in cardiac tissue. The administration of DIC sensitizers, namely Rosi and Flud, further increased the DIC incidence and altered the DOX distribution in our mouse model.

### Targeting the IFN-γ pathway or modulating the PPAR-γ pathway prevents DIC in mice

**Fig. 6:**
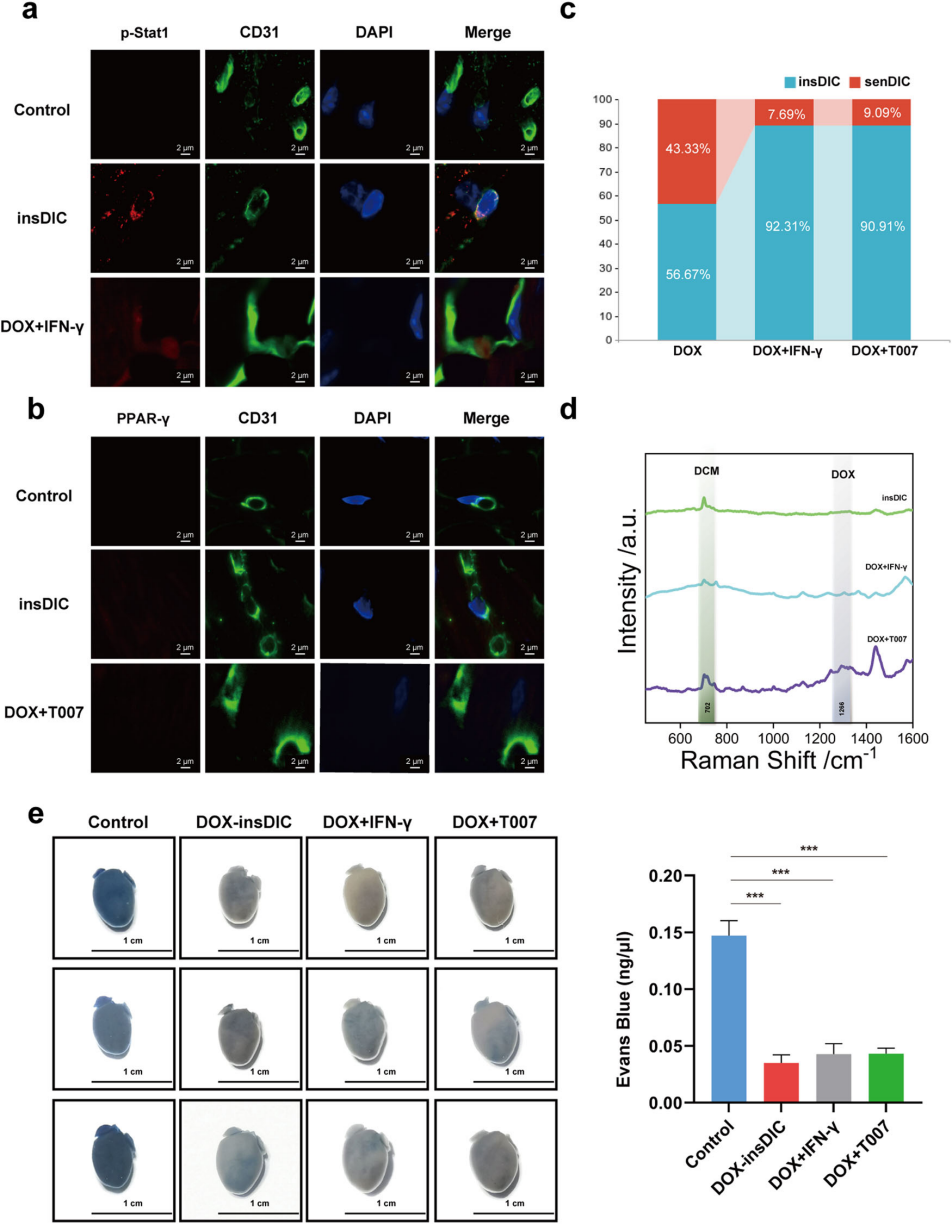
Targeting the IFN-γ pathway or modulating the PPAR-γ pathway prevents DIC in mice. **a**, Immunofluorescence staining was performed to detect the levels of p-Stat1 in CD31-labeled ECs. Scale bars, 5 µm. **b**, Immunofluorescence staining was performed to detect the expression of PPAR-γ in CD31-labeled ECs. Scale bars, 5 µm. **c**, The proportions of mice in the insDIC group and insDIC group in the DOX group, DOX+IFN-γ group and DOX+T007 group were analyzed. **d**, SERS spectra of the DOX drug concentration in heart tissues from mice in the insDIC (green line), DOX+IFN-γ (yellow line) and DOX+T007 (red line) groups. Experimental conditions: laser power of 0.95 mW (632.8 nm); 5 scans of 10 s. **e**, The overall morphology of the hearts of the control, insDIC, DOX+IFN-γ and DOX+T007 groups after Evans blue staining. ***P < 0.001. Scale bars, 1 cm. *n* = 6. All the data are presented as the mean ± s.e.m.

We conducted p-Stat1 and PPAR-γ staining on CD31-positive cardiac microvascular ECs from the insDIC group to explore potential agents that could prevent DIC in clinical practice. The staining results revealed p-Stat1-positive but PPAR-γ-negative staining (Fig. 6a, b), indicating the activation of the IFN-γ pathway but not the PPAR-γ pathway in these cells. This finding suggests a possible association between the activation of the IFN-γ pathway and the inhibition of the PPAR-γ pathway concerning DOX retention in insDIC blood vessels. We selected the recombinant IFN-γ protein as an IFN-γ pathway agonist and T007 as a PPAR-γ pathway inhibitor to identify potential therapeutic agents that could modulate DIC sensitivity and prevent DIC development. Administering these drugs to DOX-treated mice significantly increased p-Stat1 levels and inhibited PPAR-γ expression in cardiac ECs (Fig. 6a, b). Doppler echocardiography revealed that the combined administration of the recombinant IFN-γ protein or T007 significantly prevented DIC in mice (Fig. 6c). We further investigated the effects of the drugs on the DOX distribution in mice by measuring the DOX content in the serum and heart tissue. The candidate drugs resulted in increased DOX concentrations in the serum but decreased concentrations in the heart tissue (Fig. 6d). Evans blue staining revealed that the drugs hindered the uptake of exogenous substances in cardiac tissue (Fig. 6e).

Therefore, our findings suggest that these candidate agents can mitigate DIC sensitivity and prevent DIC onset. Activating the IFN-γ pathway and inhibiting the PPAR-γ pathway may represent therapeutic approaches for DIC prevention. Further research and clinical trials are needed to validate the efficacy and safety of these agents in DIC prevention.

### P-gp regulated by IFN-γ is involved in DOX transport and DIC sensitivity

**Fig. 7:**
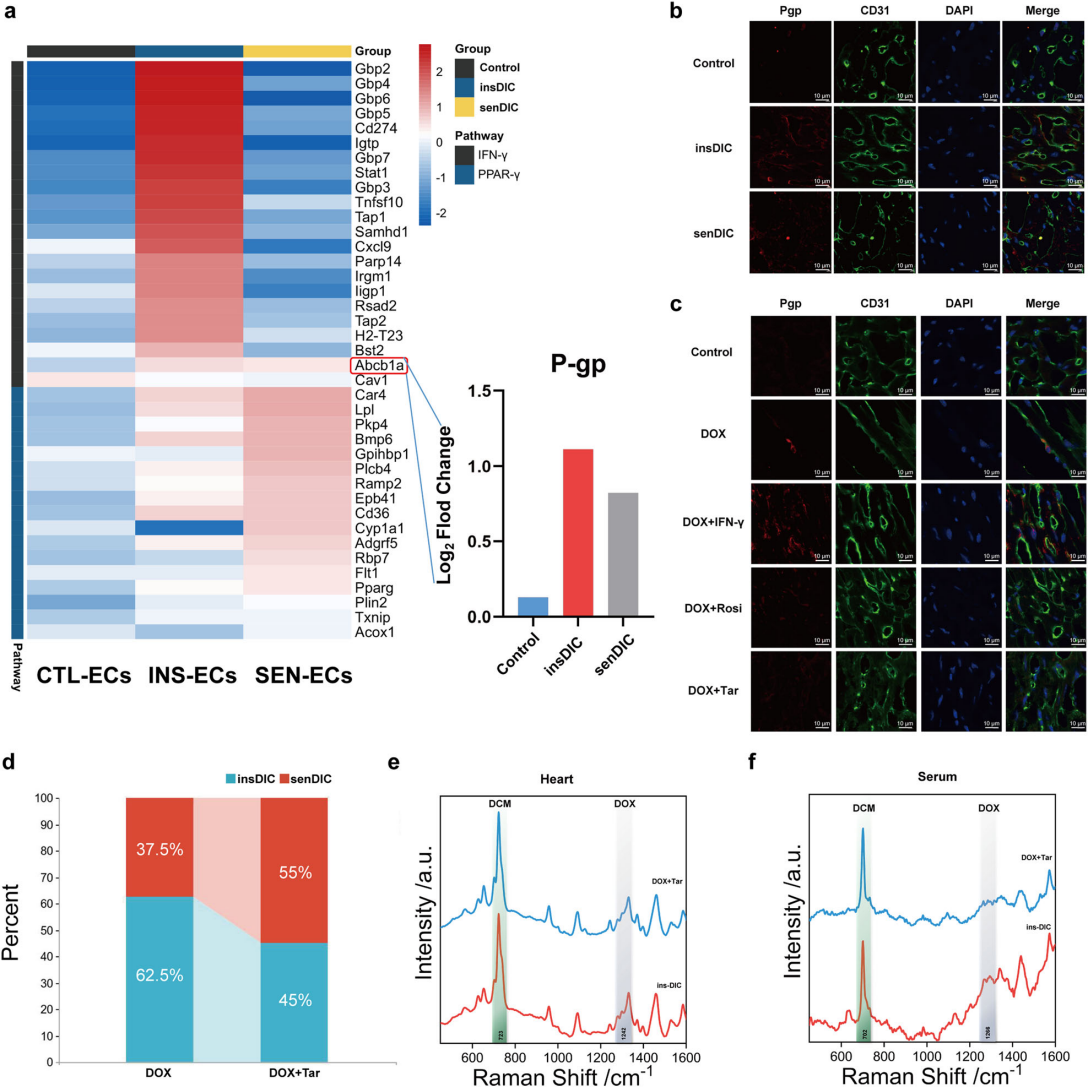
P-gp plays a vital role in DIC resistance. **a**, Heatmap analysis of genes involved in the IFN-γ and PPAR-γ pathways that were expressed in EC subsets from the CTL-EC, INS-EC and SEN-EC groups revealed P-gp (left), and changes in expression among the three groups were analyzed (right). **b**, Immunofluorescence assays of CD31-labeled ECs were performed to detect the expression of P-gp in DIC mice. Scale bars, 5 µm. **c**, Immunofluorescence assays of CD31-labeled ECs were performed to detect the expression of P-gp in different mouse models. Scale bars, 5 µm. **d**, The proportions of mice in the insDIC group and senDIC group in the DOX group and DOX+Tar group were analyzed. **e**, SERS spectra of the DOX drug concentration in heart tissues from mice in the insDIC (red line) and DOX+Tar (blue line) groups. Experimental conditions: laser power of 0.95 mW (632.8 nm); 5 scans of 10 s. **f**, SERS spectra of the serum DOX concentration in the insDIC (red line) and DOX+Tar (blue line) groups. Experimental conditions: laser power 0.95 mW (632.8 nm); 5 scans of 10 s.

The preliminary results highlight that individual differences in DIC arise primarily from variations in the drug transport capacity and DOX distribution, which are associated with the IFN-γ and PPAR-γ pathways. We conducted a heatmap analysis of DEGs related to these pathways in the EC subpopulations (EC-cmv, INS-ECs and SEN-ECs) to explore these results further. Among the genes that were differentially expressed, the only one with a role in drug transport was *Abcb1a*, which encodes P-gp (Fig. 7a). P-gp is an ATP-binding efflux transporter protein that is associated with biofilms. It is most well known for its involvement in the composition of the BBB and its ability to remove toxic substances from cells^32^. P-gp can transport multiple substrates, one of which is DOX. Through scRNA-seq, we detected higher expression levels of the P-gp mRNA in the insDIC group than in the senDIC group (Fig. 7a). We performed P-gp staining on CD31-positive ECs in the heart tissues of the three groups of mice and observed higher P-gp expression levels in the insDIC group (Fig. 7b). In addition, P-gp expression in ECs corresponded to the insDIC and senDIC groups after activation of the IFN-γ and PPAR-γ pathways. The results indicated that the recombinant IFN-γ protein upregulated P-gp expression, whereas Rosi significantly inhibited P-gp expression (Fig. 7c). We used the P-gp inhibitor Tar, which noncompetitively inhibits the binding of ATPase activity to P-gp^33^, to validate the critical role of P-gp in DIC resistance. As expected, Tar effectively suppressed P-gp expression (Fig. 7c). The combination of Tar with DOX increased the incidence of DIC (55% in the SenDIC group), which was observed on the fifth day, and all the mice died on the seventh day (Fig. 7d). This result indicates that Tar has a greater propensity to induce DIC than Rosi and Flud. We utilized SERS to measure the DOX content in mouse serum and cardiac tissue and evaluate the effect of Tar on the DOX distribution. Interestingly, Tar increased DOX accumulation in cardiac tissue while reducing its content in serum (Fig. 7e, f). These findings suggest that P-gp in ECs plays a critical role in DIC resistance and that mice are unable to withstand DIC when P-gp in ECs is inhibited. The altered drug transport capacity mediated by P-gp malfunction probably contributes to DIC development. Further studies are needed to explore the underlying mechanisms and potential strategies to mitigate DIC associated with impaired P-gp function.

### Regulation of P-gp expression and DOX transport by the PPAR-γ and IFN-γ pathways in human ECs

**Fig. 8:**
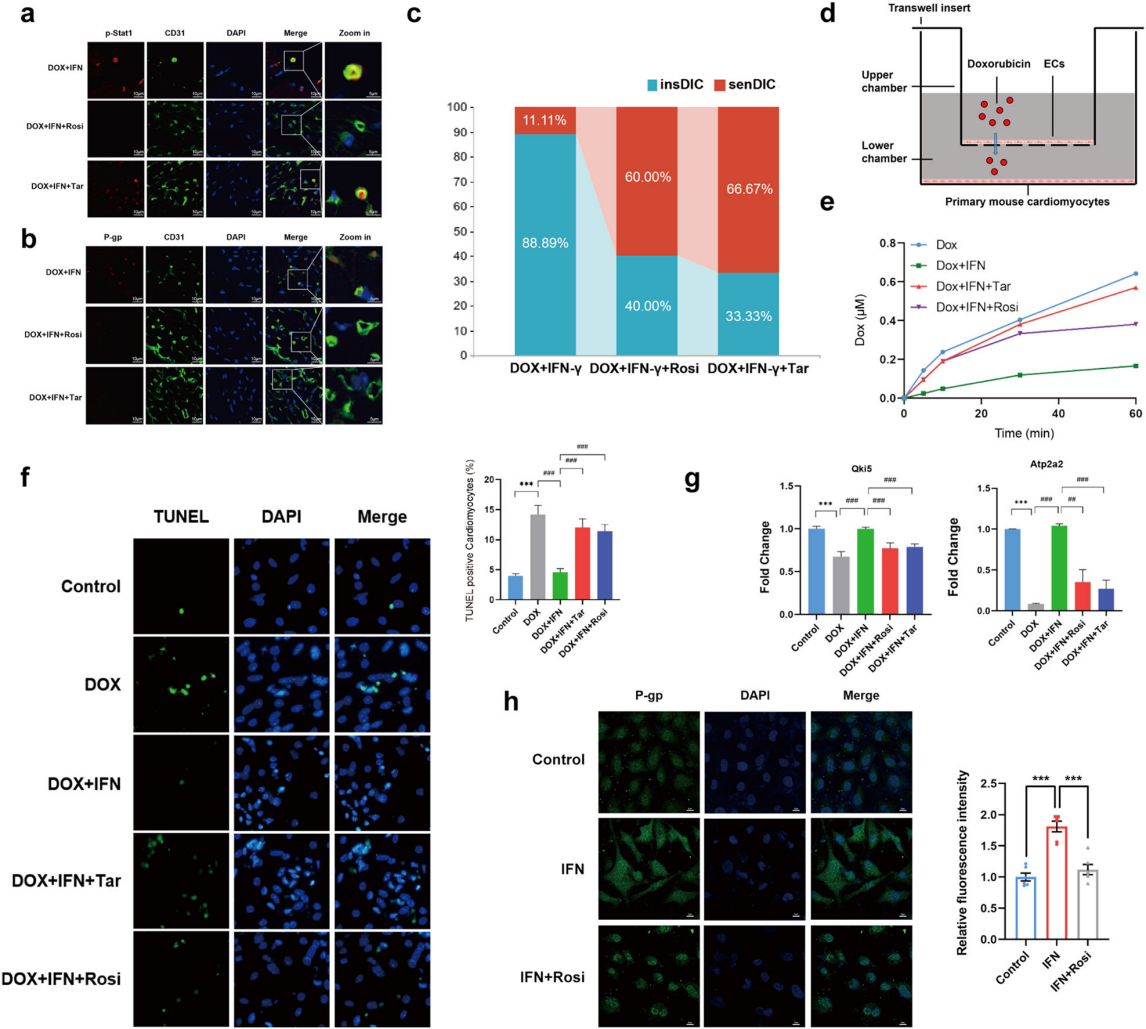
IFN-γ mediates P-gp expression and DOX transport in human ECs. **a**, Immunofluorescence staining was performed to detect the levels of p-Stat1 in the ECs from the mice in the DOX+IFN and DOX+IFN+Rosi groups. **b**, Immunofluorescence assays of CD31-labeled ECs were performed to detect the expression of P-gp in ECs from mice in the DOX+IFN and DOX+IFN+Rosi groups. Scale bars, 5 µm. **c**, The proportions of mice in the insDIC group and senDIC group in the DOX group, DOX+IFN group, DOX+IFN+Rosi group and DOX+IFN+Tar group were analyzed. **d**, Schematic of DOX drug penetration barrier model. **e**, DOX penetration in the DOX, DOX+IFN, DOX+IFN+Tar and DOX+IFN+Rosi groups was verified in a barrier model in vitro. **f**, Representative images of TUNEL staining of hearts from the control, DOX, DOX+IFN, DOX+IFN+Tar and DOX+IFN+Rosi groups. ***P < 0.001. Scale bars, 10 µm. *n* = 6. **g**, The expression of DIC markers (*Atp2a2* and *Qki5*) in the combination groups was detected via qRT‒PCR. **P < 0.01, ***P < 0.001. *n* = 6. All the data are presented as the mean ± s.e.m. **h**, Immunofluorescence staining was performed to detect the expression of P-gp in HUVECs. Scale bars, 5 µm. ***P < 0.001. All the data are presented as the mean ± s.e.m.

Activation of the PPAR-γ pathway has been shown to inhibit the IFN-γ pathway in previous studies^34^. We investigated the associations among these pathways in DIC by administering DOX, the recombinant IFN-γ protein and Rosi simultaneously. We then detected the activation of the IFN-γ pathways and the expression of P-gp. The results showed that the recombinant IFN-γ protein significantly increased p-Stat1 and P-gp levels, whereas the addition of Rosi downregulated p-Stat1 and P-gp levels (Fig. 8a, b). Doppler echocardiography revealed that the combined administration of the recombinant IFN-γ protein and Rosi led to an increased incidence of DIC (60% in the senDIC group) (Fig. 8c). The increase in DIC induced by Rosi is comparable to that of Tar, indicating that PPAR-γ pathway activation is associated with P-gp inhibition (Fig. 8a–c). These findings suggest that the activation of the PPAR-γ pathway inhibits the DIC resistance induced by IFN-γ pathway activation, causing cardiotoxicity in mice. Thus, activation of the PPAR-γ pathway may reduce P-gp expression, decrease DOX efflux and contribute to DIC by inhibiting the IFN-γ pathway. This may represent a potential molecular mechanism by which the blood–myocardium barrier exerts its function.

We constructed an in vitro barrier model to elucidate the mechanism of the blood‒myocardium barrier. A single layer of human umbilical vein ECs (HUVECs) was seeded in a Transwell chamber, while primary CMs were plated in the lower chamber. Normal saline, IFN-γ, IFN-γ combined with Tar, and IFN-γ combined with Rosi were added to the Transwell chamber in advance to establish the DOX drug penetration barrier model (Fig. 8d). Then, 1 μM DOX was added to the upper chambers, and the lower solution was collected and detected at 5, 10, 30 and 60 min. The results showed that activation of the IFN-γ pathway notably reduced the penetration of DOX, indicating the establishment of a drug barrier (Fig. 8e). The combined use of Rosi or Tar with IFN-γ significantly increased DOX penetration, indicating that both Rosi and Tar disrupted the drug barrier formed by the IFN-γ pathway (Fig. 8e). We used primary CMs from the lower chamber of the in vitro model for subsequent experiments to further investigate the impacts of the IFN-γ and PPAR-γ pathways on DIC-mediated damage. Our results revealed that DOX administration led to significantly increased CM damage, whereas activation of the IFN-γ pathway in ECs mitigated the occurrence of damage. However, when the PPAR-γ pathway was activated or P-gp was inhibited, this protective effect was disrupted and the damage was exacerbated (Fig. 8f, g). Moreover, our in vitro studies in HUVECs indicated that IFN-γ promoted P-gp expression, whereas this effect was blocked when the PPAR-γ signaling pathway was activated by Rosi (Fig. 8h). Simultaneously, we treated HUVECs with si-STAT1 and si-PPAR-γ. We found that P-gp expression was obviously downregulated after STAT1 knockdown but upregulated after PPAR-γ knockdown (Supplementary Fig. 4). These results indicate that IFN-γ reduces DOX penetration and provides resistance to DIC by promoting P-gp expression both in vivo and in vitro. The PPAR-γ pathway downregulates P-gp expression by inhibiting the IFN-γ pathway, leading to increased DOX penetration and the development of DIC.

## Discussion

This study revealed the underlying mechanisms of differences in DOX drug transport and sensitivity to DIC. We found that insensitivity to DIC is related to reprogramming ECs to regulate DOX drug transport. Differences in P-gp expression are an important cause of sensitivity to DIC. Activation of the IFN-γ pathway reduces DOX penetration and prevents DIC by upregulating P-gp. Activation of the PPAR-γ pathway enhances DOX penetration and causes DIC by blocking the ability of IFN-γ to upregulate P-gp. This study revealed that sensitivity to DIC is related to the reprogramming of ECs induced by IFN-γ, providing a new perspective for addressing DIC.

Although DIC has been extensively investigated, the pathogenesis of DOX-related heart injury remains poorly understood^24^. The primary mechanisms contributing to CM injury induced by DOX include the excessive production of reactive oxygen species, resulting in lipid, DNA and protein damage; damage to TOP2B, leading to DNA double-strand breaks; and mitochondrial damage^35^. In addition, recent reports suggest that DOX may influence histone by interacting with chromatin topologically associating domains^36^, histone abnormalities in CMs are linked to the progression of heart failure^37^. Furthermore, DOX induces various forms of cell death in CMs, including autophagy^1^, ferroptosis^38^, necroptosis^39^, pyroptosis^40^ and apoptosis^41^. However, current research has focused mainly on the mechanisms of heart damage caused by DOX, with a limited understanding of the potential protective mechanisms in patients who use DOX without experiencing DOX-related heart damage. We constructed a mouse model to investigate the molecular mechanisms underlying resistance to DIC. Our findings demonstrate that these mice exhibit DIC sensitivity, similar to the observed clinical phenomenon. Considering that anthracyclines share similar DIC mechanisms, our model can also provide insights into the side effects associated with anthracyclines and other cardiotoxic drugs.

ECs play a role in the body’s physiological homeostasis to ensure the transport of nutrients, control vascular permeability and regulate vascular tone, which are indispensable parts of the circulatory system^42^. ECs are divided into arterial ECs, venous ECs, microvascular ECs, lymphatic ECs and endocardial cells^31^. We found reprogramming of microvascular ECs after the DOX injection (Fig. 4) and differences in the distribution of the drug in blood vessels and heart tissue between different groups of mice (Fig. 2a–f). First, vascular integrity and tight junctions between ECs in the senDIC group were affected by DOX toxicity, but electron microscopy revealed no changes in vascular integrity or tight junctions in the three groups of mice (Fig. 2g). Therefore, we speculate that cardiac ECs might form an ‘immunoactivated barrier’ to prevent DOX present in the blood of mice in the insDIC group from entering the heart, which is similar to the process of discovering the BBB with aniline dye^43^. We chose Evans blue to prove the existence of a barrier in the hearts of the mice in the insDIC group, and we speculated that this barrier might be the blood‒myocardium barrier. Although the concept of the blood‒myocardium barrier was proposed as early as 1996, few in-depth study of the existence and mechanism of the blood‒myocardium barrier has been conducted, and the blood‒myocardium barrier is still only at the conceptual level^44^. The reasons are as follows: (1) The blood‒myocardium barrier is not activated under normal conditions and is difficult to detect. (2) The blood‒myocardium barrier has no anatomical basis, unlike other barriers, and thus it cannot be directly observed; by contrast, the BBB is composed of ECs, pericytes, capillary basement membrane and astrocyte endfeet^45^. (3) Sampling heart tissue from a living body is difficult, and tissue is often obtained after death, which greatly affects the specific situation of the physiological state. Therefore, the blood‒myocardium barrier has not been adequately tested. Nevertheless, an in-depth study of the blood‒myocardium barrier and the mechanism that regulates the on/off switch of this barrier has significant clinical applications, including the promising application of the blood‒myocardium barrier to avoid the side effects of cardiotoxic drugs in the future. For example, the use of cardiotoxic drugs with a blood‒myocardium barrier activator can prevent these drugs from entering cardiac tissues, thereby attenuating cardiotoxicity while maintaining their efficacy, such as with anthracycline or fluorouracil^46^. Our study may be the first to experimentally confirm the existence of a blood‒myocardium barrier, but more evidence is needed to support this idea. The study of the blood‒myocardium barrier provides theoretical support for understanding the heart microstructure and reducing cardiotoxic drug side reactions.

IFN-γ has been shown to be secreted primarily by activated lymphocytes, such as CD4^+^ and CD8^+^ T cells^47^. IFN-γ has a variety of functions, including regulating antigen presentation, promoting inflammation and signaling, and activating and polarizing macrophages^48^. IFN-γ can upregulate the expression of P-gp and can also change the localization of P-gp to promote its transport activity^49^. Moreover, P-gp is an important component of the BBB^14^, but whether IFN-γ can reduce DOX penetration through the upregulation of P-gp expression has not been reported. We demonstrated in vivo that the activation of the IFN-γ pathway attenuates DOX penetration by administering activators and inhibitors to our knowledge of the IFN-γ pathway and showed that this process can be blocked by the P-gp inhibitor Tar. Therefore, the main reason for resistance to DIC is that P-gp is upregulated after the activation of the IFN-γ pathway, thus activating the drug efflux function of P-gp, preventing DOX from entering cardiac tissue from the blood and protecting the heart. The PPAR-γ pathway has been reported to inhibit the IFN-γ pathway^50^. By combining Rosi and the recombinant IFN-γ protein, we confirmed that the IFN-γ pathway was inhibited due to the activation of PPAR-γ in the senDIC group, thus downregulating P-gp expression and increasing DOX penetration. DOX is able to enter the heart from the blood unimpeded and promote DIC.

We administered DOX to mice at the same dosage, but the reason for dividing them into two groups, insDIC and senDIC, based on different cardiac functions was the main focus of our study. We found that the insDIC group was produced because INS-ECs, which are characterized by activation of the IFN-γ pathway, decrease DOX penetration, but we did not investigate how IFN-γ is released to activate INS-ECs. We hypothesize that DOX-induced inflammatory responses lead to an enrichment of macrophages, which present antigens and recruit T cells to release IFN-γ to cardiac ECs, thereby reducing DOX penetration. However, this process was blocked in the senDIC group by activation of the PPAR-γ pathway, which increased the DOX penetration capacity. The mechanism by which PPAR-γ is activated in the senDIC group is unknown. We speculated that, due to the heterogeneity among different mice, some mice expressed genes that promote PPAR-γ expression, whereas other mice did not. This hypothesis also explains why the mice were obviously divided into two groups after the DOX injection, which requires further study. Moreover, the recombinant IFN-γ protein and T007 were found to significantly increase the survival rate and cardiac systolic function of mice after the DOX injection. However, experimental results verifying the effects of these two drugs combined with DOX on reducing cardiac toxicity and other physiological indicators are still lacking. DIC can also lead to prolonged QT intervals and arrhythmia^51^. This study focused only on indicators related to heart damage, and parameters related to DIC should be evaluated in a more comprehensive manner. Moreover, if we want to identify drugs that can meet the clinical application criteria and target the IFN-γ and PPAR-γ pathways, we need to confirm whether the drugs themselves have other side effects and multiorgan targets and strictly monitor the dose of drugs used and the physiological indicators of patients. In addition, ensuring that the combination of drugs does not affect the antitumor activity of DOX itself is something that we need to address in the clinical translation. We used scRNA-seq mining and found that EC reprogramming is the key to DIC sensitivity, but whether other cells also play important roles has not been explored in detail.

Mice are the most commonly used model for DIC studies, induced mainly by i.p. injection and caudal intravenous injection. However, a caudal intravenous injection increases the risk of phlebitis and tail rot in mice, increasing the likelihood of death and infection. By contrast, abdominal injection is easier to perform, less risky and more suitable for establishing a DIC model^1^. Sex dose not affect DIC in mice^52^. We excluded the influence of hormonal changes in female mice by selecting male mice to study their sensitivity to DIC. In terms of pharmacokinetics, DOX metabolic processes are similar in mice and humans, and DOX is metabolized through bile in both species^53^. It was previously reported that the concentration of DOX in clinical patients is approximately 5.9 mg/l (ref. ^54^). In our study, based on SERS detection (Fig. 2f), the concentration of DOX in mice is approximately 1–7 mg/l (ref. ^29^). In addition, the proportion of cardiotoxicity in our mouse model was 68.96% (Fig. 1c), and the proportion of cardiotoxicity in clinical patients was 51.37% (Supplementary Scheme 1), which was similar. Simultaneously, we conducted long-term maintenance of the model mice and echocardiographic examinations. The results indicated that, starting on day 14 postinjection, no deaths were observed in the senDIC group, and their heart function gradually returned to normal levels by day 28. In addition, the heart function of the mice in the insDIC group remained stable regardless of the duration of housing, further confirming the reliability of our groups (Supplementary Fig. 5). Therefore, our established mouse model of DIC sensitivity can partially simulate the clinical situation of patients using DOX. In summary, we found that the mice in the insDIC group that received the same dose of DOX did not suffer from DOX-related heart injury, and DOX penetration was reduced, which resulted in DOX being retained in the blood and less of the drug entering the heart tissue, avoiding the occurrence of DIC. We subsequently performed scRNA-seq and found that the reason for the resistance to DIC was the reprogramming of ECs, which caused microvascular CTL-ECs to differentiate into INS-ECs upon the activation of the IFN-γ pathway, resulting in DOX retention in blood vessels. Activation of the PPAR-γ pathway promoted CTL-ECs differentiation into SEN-ECs and DOX enrichment in the heart. Through bioinformatics analysis and multiple drug combination models, we found that differences in P-gp expression were an important cause of DIC sensitivity in ECs and that IFN-γ upregulated P-gp expression to promote DOX efflux and resist DIC. PPAR-γ inhibited P-gp expression by blocking the IFN-γ pathway to increased sensitivity to DIC. Moreover, we verified the mechanism of the in vivo model by establishing an in vitro barrier model and obtained consistent conclusions. Our findings provide new insights into the mechanism of adverse reactions to cardiotoxic drugs such as DOX, as well as a new perspective for developing a feasible plan to reduce the rate of adverse reactions to cardiotoxic drugs that can be applied clinically.

## Supplementary information

Supplementary information
