# Supplementary material for: IFN-γ reprograms cardiac microvascular endothelial cells to mediate doxorubicin transport and influences the sensitivity of mice to doxorubicin-induced cardiotoxicity
Source: Exp Mol Med. 2025 Jan 22;57(1):249–63. doi: 10.1038/s12276-024-01389-7 (PMC11799190; doi:10.1038/s12276-024-01389-7)
Supplement: Supplementary file 1 — Supplementary information [file 12276_2024_1389_MOESM1_ESM.pdf]

SUPPLEMENTAL MATERIAL

IFN-γ Reprogramming Cardiac Microvascular Endothelial Cells to Mediate Doxorubicin Transport Determines Cardiotoxicity Sensitivity in Mice

EXPANDED METHODS

Echocardiographic measurements

Transthoracic echocardiography was performed to evaluate cardiac function of mice by echocardiography imaging using the Vevo2100 High-Resolution Imaging System (Visual Sonics, Toronto, ON, Canada) equipped with a 10-MH2 phased-array transducer with the M-mode recording. Mice were anaesthetized with by Avertin (200 mg/kg i.p., Sigma-Aldrich, St Louis, USA) and the anterior chest area was depilated using NairTM depilatory cream (Church & Dwight Co., Inc., Princeton, NJ, USA).They were then positioned on an electric heating Pad (part of the Visual Sonics Vevo Integrated Rail System II) to maintain body temperature at 37°C. Medical ultrasound gel (Tianjin Yajie Medical Material Co., Ltd., Tianjin, China) was used as a coupling agent between the ultrasound scan-head and the skin. Two-dimensional targeted M-mode traces were recorded from the parasternal short-axis view at the level of the mid-papillary muscles and from the parasternal long-axis view at the level of immediately under of the papillary muscle. The left ventricular parameters including left ventricular end diastolic volume (LVEDV), left ventricular end systolic volume (LVESV), left ventricular internal dimension at end diastole (LVIDd), and left ventricular internal dimension at systole (LVIDs) were measured based on

M-mode recordings. The data are presented as the average of measurements of three consecutive beats. EF was calculated as (LVEDV-LVESV)/LVEDV×100% and FS as (LVIDd-LVIDs)/LVIDd×100%, respectively.

TUNEL assay

Heart tissues were embedded with paraffin and cut into thin sections (4 μm) were fixed and labeled using the In Situ Cell Death Detection Kit (Roche, Mannheim, Germany) following the manufacturer’s instructions to assess apoptosis. After TUNEL staining, the nuclei were counterstained with DAPI (Abcam, USA). The TUNEL-positive cells were observed under a fluorescence microscope (Zeiss, Jena, Germany) and the ratio of TUNEL-positive cells to DAPI-positive nuclei were calculated.

Immunofluorescence staining

Immunostaining of cardiac myocytes was performed for detection of following proteins: P-gp (Cat#ab235954, Abcam, USA), PPAR-γ (Cat#2443, CST, USA), P-stat (Cat#9167, CST, USA), CD31 (Cat#ab24590, Abcam, USA), DAPI (Cat#ab104139, Abcam, USA). Secondary antibodies conjugated to fluorophore (Alexa Fluor 488 and 594, Molecular Probes Inc) were used to visualize the target proteins.

Cell cultures

HUVEC were cultured in DMEM High Glucose GlutaMAX (Gibco, Carlsbad, CA) supplemented with 10% FBS, 5 mM penicillin/streptomycin (Gibco, Carlsbad, CA) and incubated under 5% CO2 at 37°C.

Transmission electron microscopy analysis

Hearts were harvested from WT and KD mice, in basal conditions and 7 days after DOX treatment. 1 mm^3 heart pieces from the left ventricular wall were fixed in 1.25% glutaraldhehyde (v:v in 0.1 M sodium cacodylate, pH 7.2) overnight at 4°C. Heart samples were washed in 0.1 M sodium cacodylate (3 times for 30 minutes), post-fixed and thin sections were imaged on a Tecnai-20 electron microscopy (Philips-FEI, Hillsboro, Oregon).

Surface-enhanced Raman spectroscopy

Serum samples and tissue suspension to be measured were taken, and then six times the volume of methanol solution was added to the sample to avoid light. The sample was flipped and shaken repeatedly until a large amount of red flocculent precipitate was formed in the sample. The samples were subsequently centrifuged at 4℃ for 12,000 rpm 20 min. Remove the supernatant into a 4 mL EP tube, place the EP tube open in a water bath, tilt, and heat at 70℃. At the same time, the sample is blown repeatedly with a hair dryer to accelerate its evaporation until there is no liquid at all. The concentration was then redissolved with 1 μL water and the tube until fully dissolved. Calibrating the machine prior to detection, we examined the sample using a

633 wavelength laser. Samples were then configured at the ratio of 20 μL of silver nanoparticles, 10 μL of the sample, 2 μL of dichloromethane and 2.3 μL of calcium chloride. Samples were measured with a scanning confocal Raman microscope, and the sample drug concentration was calculated against a standard curve.

Quantitative real-time PCR

Total RNA was extracted from CMs or myocardium using TRIzol reagent (Invitrogen, Carlsbad, CA, USA). NANODrop2000c (Thermo Scientific, Carlsbad, USA) was utilized to verify the quality of RNA samples. Total RNA was reverse transcribed into cDNA using the Trans-Script All in-one First-strand cDNA Synthesis Super mix for qPCR Kit (TransGen Biotech, Beijing, China). The ABI 7500 fast Real-Time PCR system (Applied Biosystems, Foster City, USA) was used to measure the expression levels of mRNAs by using SYBR Green (Roche, Basel, Basel-City, Swiss). The primer sequences utilized for RT-PCR analysis are listed in **Supplementary Table 1**.

Isolation and culture of neonatal mouse ventricular cells (NMVCs)

Briefly, after rinsed in the PBS, the isolated hearts of neonatal mice were cut into pieces and digested in 0.25% trypsin. The obtained NMVCs were cultured in Dulbecco's modified Eagle medium (DMEM, Hyclone Laboratories, Utah, USA) supplemented with 10% fetal bovine serum (Gibco, California, USA), 100 U/mL penicillin and 100 μg/mL streptomycin.

Evans blue dye

200 μL of 2% Evans blue (Solarbio, Beijing, China) was injected through the Abdominal vein into the mice. After 5 min, the mice were detached and the heart, liver, and brain were quickly removed. The hearts was irrigated three times with normal saline, and then were photographed. Tissues was ground and homogenized, added to acetone according to the ratio of tissue: acetone 3:7, incubated at room temperature for 24 h and centrifuged at 13,500 rpm for 15 min. 200 μL of liquid supernatant was added to a 96-well plate and the absorbance was measured at 620nm.

Detection of mouse IFN-γ level

Heart IFN-γ concentration was detected using Mouse IFN-γ ELISA Kit (Elabscience, Wuhan, China) according to the manufacturer's instructions. Following the addition of stop solution, the absorbance of each sample was measured spectrophotometrically at a wavelength of 450 nm using a microplate reader (BioTek, Richmond, VA, USA).

Western blotting

An equivalent amount of protein from cells extracts was resolved on SDS–PAGE gels, after which the proteins were transferred to a nitrocellulose membrane. The nitrocellulose membranes with transferred protein were blocked (5% bovine serum albumin) and probed with the following primary and secondary antibodies: P-gp (Cat#ab170904, Abcam, USA), PPAR-γ (Cat#2443, CST, USA), P-stat (Cat#9167, CST, USA), GAPDH (Cat#AB0037, Abways Technology, China). IRDye 800CW

goat anti-rabbit IgG secondary antibody (Alexa Fluor 488, Cat#926-32211, Licor , USA) were used to visualize the target proteins.

Single cell sequencing

Single-cell RNA sequencing (scRNA-seq) was employed to investigate the gene expression profiles of mice heart. Fresh cardiac tissues were dissociated into single-cell suspensions using enzymatic digestion and mechanical separation. The cells were then washed, resuspended, and loaded onto a microfluidic chip for droplet-based scRNA-seq library preparation. The 10x Genomics Chromium platform was used for single-cell encapsulation and barcoding, followed by reverse transcription and cDNA amplification. The cDNA libraries were sequenced on an Illumina NovaSeq platform.

Data analysis was performed using the Cell Ranger software pipeline provided by 10x Genomics. Read alignment was conducted against the mouse reference genome (mm10) to generate gene-cell count matrices. Quality control steps were employed to filter cells with low transcript counts and detect potential doublets. The data was normalized using a global-scaling normalization method, and highly variable genes were identified for subsequent analysis. Dimensionality reduction was performed using principal component analysis (PCA) and t-distributed stochastic neighbor embedding (t-SNE) to visualize the cellular heterogeneity.

To identify differentially expressed genes (DEGs) between groups, we employed the negative binomial test implemented in the Seurat package. GO and KEGG

analysis were conducted to assess enrichment of specific gene pathways and functions. Additionally, we used Monocle 3 to perform pseudo-time trajectory analysis and identify dynamic gene expression patterns during endothelial cell differentiation.

Figure Legends

Supplementary Scheme 1

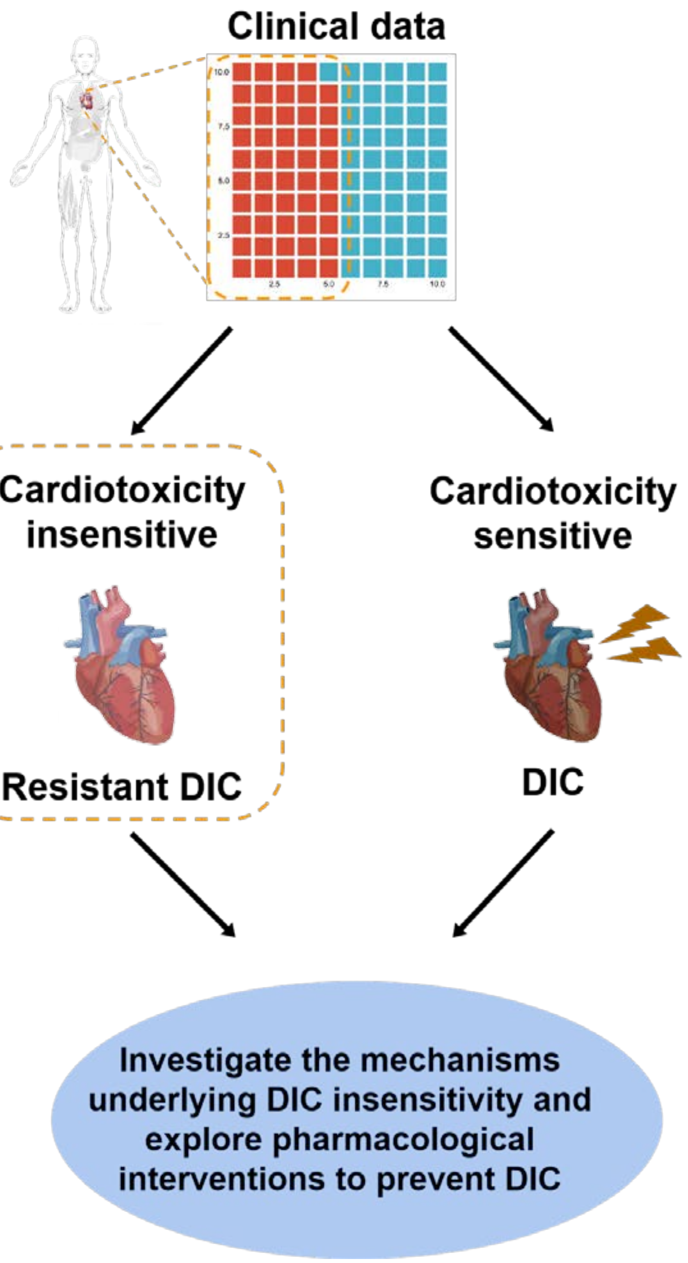

Supplementary Scheme 1. The sensitivity of patients to DIC exhibited variations, as depicted in the schematic illustration.

Supplementary Fig. 1.

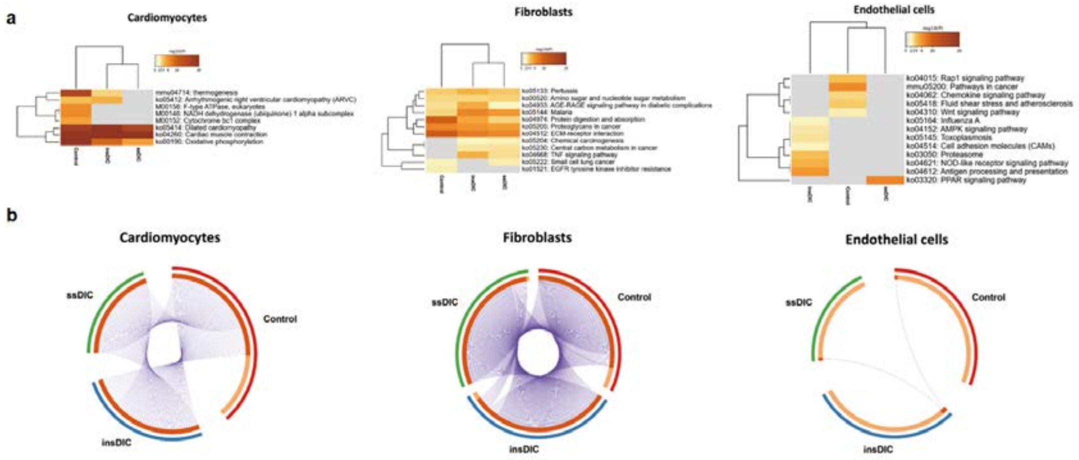

Supplementary Fig. 1. (a) KEGG analysis was performed according to the principle of FDR<0.01 and $|\log_2$fold Change$|>1$ to filter out differentially expressed genes were screened from the transcriptional profiles of the three groups of CMs, fibroblasts and ECs. **(b)** The correlation ring plot shows the correlation of the transcriptome among the three groups of mouse CMs, fibroblasts and ECs.

Supplementary Fig. 2.

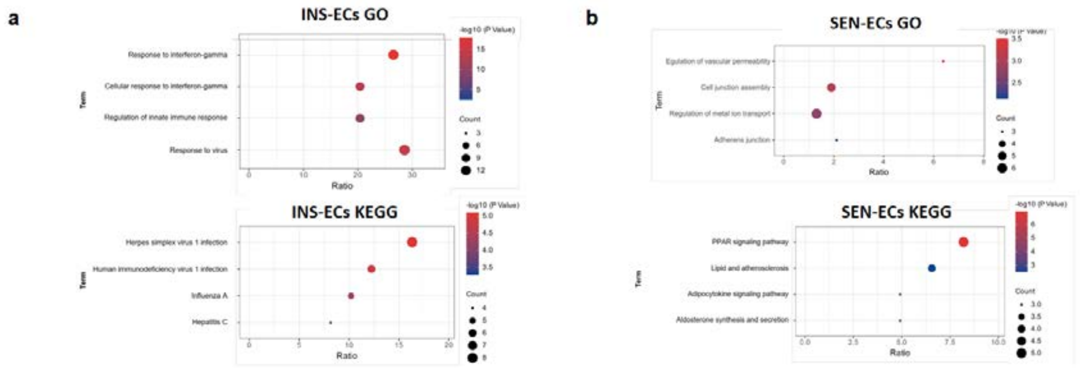

Supplementary Fig. 2. (a) GO and KEGG analysis was performed according to the principle of FDR<0.01 and $|log_2$fold Change$|>1$ to filter out differentially expressed genes were screened from the transcriptional profiles of the three groups of INS-ECs. **(b)** GO and KEGG analysis was performed according to the principle of FDR<0.01 and $|log_2$fold Change$|>1$ to filter out differentially expressed genes were screened from the transcriptional profiles of the three groups of SEN-ECs.

Supplementary Fig. 3.

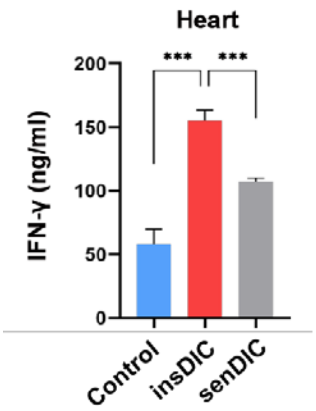

Supplementary Fig. 3. IFN-γ content in heart of mice in different groups by ELISA. ****P* < 0.001. n=6. All data are presented as mean ± SEM.

Supplementary Fig. 4.

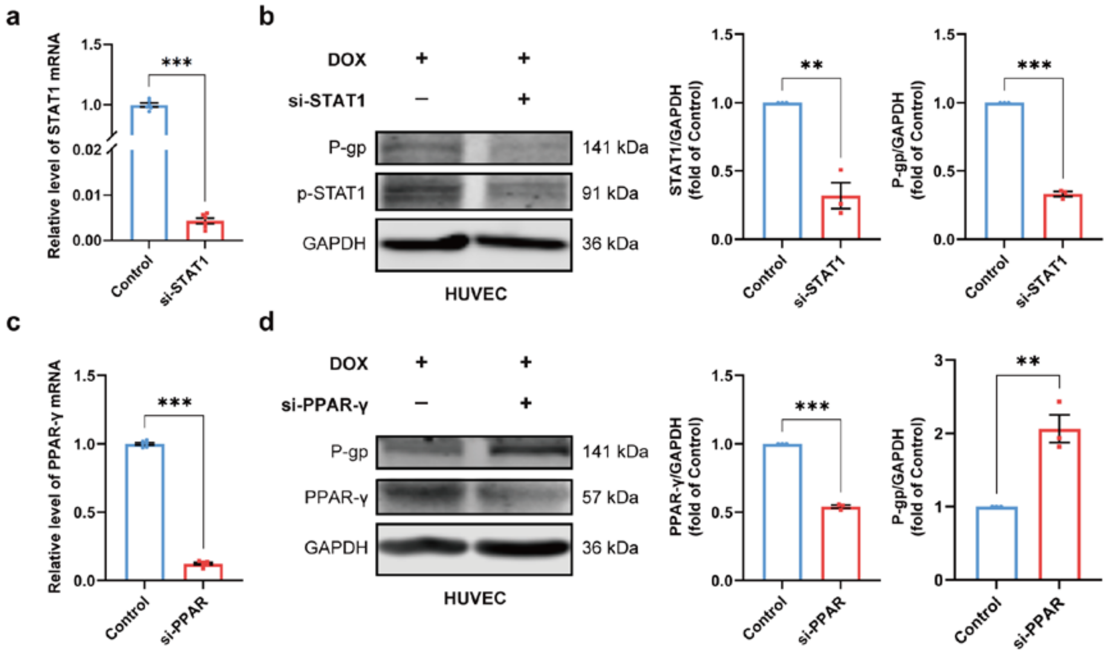

Supplementary Fig. 4. (a) HUVECs were treated with si-STAT1, and the expression of STAT1 mRNA was detected using qRT-PCR. ****P* < 0.001. n=6. **(b)** Representative immunoblot (left) and relative quantification (right) of P-gp and p-STAT1 expression. HUVEC cells were treated with si-STAT1 and subsequently exposed to doxorubicin. Cell extracts were then collected for detection. ***P* < 0.01, ****P* < 0.001. n=3. **(c)** HUVECs were treated with si-PPAR-γ, and the expression of PPAR-γ mRNA was detected using qRT-PCR. ****P* < 0.001. n=6. **(d)** Representative immunoblot (left) and relative quantification (right) of P-gp and PPAR-γ expression. HUVEC cells were treated with si-PPAR-γ and subsequently exposed to doxorubicin. Cell extracts were then collected for detection. ***P* < 0.01, ****P* < 0.001. n=3. All data are presented as mean ± SEM.

Supplementary Fig. 5.

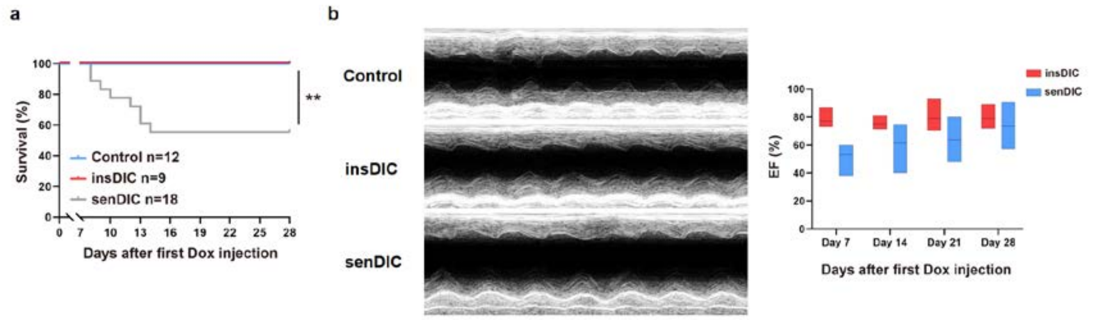

Supplementary Fig. 5. (a) Kaplan–Meier long term of survival curves of mice pretreated with saline (Control), or 12.3 mg/ml (clinical doses) DOX (i.p.) on day 0 and day 3. **(b)** Representative echocardiographic photographs showing Control, insDIC and senDIC cardiac function in long term of mice. (n=15 mice per group). The proportion of mice in insDIC group and senDIC group were analyzed.

Supplementary Table 1.

Mouse Atp5a1- F	GAAATCTCCATGCCTCTAAC
Mouse Atp5a1- R	TTCCCAAACACGACAAC
Mouse Esrra- F	GGGCACAAGGAGGAGG
Mouse Esrra- R	CATTGGAGGCCGGACAG
Mouse Sdha- F	GCTCATCGGTGTTGCTG
Mouse Sdha- R	TTTGCTCTTATTCGGTG
Mouse Atp2a2- F	TGGTAGCCAATGCAATCGTG
Mouse Atp2a2- R	CACTTTGCCCATTTCAGGCT
Mouse Qki5- F	TGTGTTAGGTGCGGTGGCTA
Mouse Qki5- R	TGTGTTAGGTGCGGTGGCTA
Mouse miR-330-5p- F	TCTCTGGGCCTGTGTCTTAGGC
Mouse miR-330-5p- R	ATCCAGTGCAGGGTCCGAGG
Mouse Lnc-Mhrt- F	ACACGGCGTTCTTGAGTTT
Mouse Lnc-Mhrt- R	AGTATGAGGAGTCGCAGTCG
Mouse GAPDH-F	ACTGAGGACCAGGTTGTC
Mouse GAPDH-R	TGCTGTAGCCGTATTCATTG
Stat1-F	CAGCTGAACATGTTGGGAGAG
Stat1-R	CTCGCTCCTTGCTGATGAAG
PPAR-γ-F	TTAGATGACAGCGACTTGG
PPAR-γ-R	GGACTCAGGGTGGTTCAG
si-STAT1	CCAUGGAAAUCAGACAGUA
si-PPAR-γ	UGGAGUUCAUGCUUGUGAA
